# A randomized controlled trial to compare short-term outcomes following infragastric and infracolic omentectomy at the time of primary debulking surgery for epithelial ovarian cancer with normal-appearing omentum

**DOI:** 10.1186/s13048-024-01401-8

**Published:** 2024-04-19

**Authors:** Xuhui Dong, Lei Yuan, Ruoyao Zou, Liangqing Yao

**Affiliations:** 1 Department of Obstetrics and Gynecology, Shanghai First Maternity and Infant Hospital, 2699 Gaoke West Road, Pudong New District, Shanghai, People’s Republic of China; 2 Department of Obstetrics and Gynecology, Obstetrics and Gynecology Hospital of Fudan University, 128 Shenyang Road, Yangpu District, Shanghai, People’s Republic of China; 3 Department of Obstetrics and Gynecology, Guangzhou Women and Children’s Medical Center, 318 Renmin Middle Road, Yuexiu District, Guangzhou, People’s Republic of China

**Keywords:** Epithelial ovarian cancer, Infracolic omentectomy, Infragrastric omentectomy, Short-term outcomes, Omental metastases

## Abstract

**Background:**

Omentectomy is an important procedure in surgery for epithelial ovarian cancer, but the scope of omentectomy is not recommended in the guidelines. This study was performed to evaluate the benefits and risks of infragastric omentectomy in patients with epithelial ovarian cancer.

**Methods:**

This trial is a single center prospective study. Primary epithelial ovarian cancer patients with normal-appearing omentum were randomly assigned to either the control or experimental group and underwent infracolic or infragastric omentectomy, respectively. The primary endpoint was progression-free survival. This trial is registered on Chinese clinical trial registry site (ChiCTR1800018771).

**Results:**

A total of 106 patients meeting the inclusion criteria for ovarian cancer were included during the study period. Of these, 53 patients underwent infracolic omentectomy, whereas 53 patients received infragastric omentectomy. Multivariate analysis revealed that infragastric omentectomy could improve the detection rate of omental metastases (OR: 6.519, *P* = 0.005). Infragastric omentectomy improved progression-free survival significantly for those cases with higher than stage IIB disease (HR: 0.456, *P* = 0.041). Based on the short-term results, infragastric omentectomy did not cause more perioperative complications.

**Conclusions:**

Compared with infracolic omentectomy, infragrastric omentectomy may be a more appropriate surgical procedure for stage IIB-IIIC epithelial ovarian cancer patients with normal-appearing omentum.

**Supplementary Information:**

The online version contains supplementary material available at 10.1186/s13048-024-01401-8.

## Introduction

Ovarian cancer is a malignant tumor of the female genital system with the highest mortality rate, which greatly affects women’s health [1–3]. The most common histological type is epithelial ovarian cancer [4]. The omentum is one of the most common metastatic sites of epithelial ovarian cancer. Omentectomy is an important procedure in surgery for epithelial ovarian cancer, whereas infracolic omentectomy is most common [5].

According to the NCCN guidelines, omentectomy should be performed if epithelial ovarian cancer is apparently confirmed to occur in the ovary or pelvis [6]. However the scope of omentectomy was not mentioned in the guidelines. In addition, ECOG guidelines recommend that at least infracolic omentectomy is necessary during the surgery [7]. Patients who receive infracolic omentectomy may still have microscopic omental tissue residues. If omissive metastases are present, it may cause adverse effects on the diagnosis of the patient. On the other hand, residual lesions after surgery are one of the most important factors affecting the prognosis of patients [8, 9]. Moreover, residual omentum is also a common site for tumor recurrence [10]. The response of omental metastases to chemotherapy is an independent predictor of death due to ovarian cancer [11]. In addition to noted advantages, expanding the surgery scope may cause more complications such as hemorrhage and injury. At present, no research has compared the benefits and risks between infracolic and infragastric omentectomy.

Therefore, the purpose of this study was to evaluate the diagnostic value, prognosis and complications of infragastric omentectomy in epithelial ovarian cancer patients with negative intraoperative omental findings.

## Methods

This trial was an investigator-initiated, single-center, parallel-group, randomized, superiority, feasibility trial that was registered on the Chinese clinical trial registry site (ChiCTR1800018771). Our clinical trial has obtained Institutional Review Board from Ethics Committee of Obstetrics and Gynecology Hospital of Fudan University (IRB approval number: 2018-17). After Institutional Review Board approval, all patients with epithelial ovarian cancer who met certain criteria at the Obstetrics and Gynecology Hospital of Fudan University from December 2018 to February 2021 were included. The inclusion criteria for our study were (i) newly diagnosed primary epithelial ovarian cancer patients; (ii) ECOG 0–1; and (iii) no surgical contraindication. The exclusion criteria were (i) patients who found suspicious omental involvement during the surgery; (ii) patients who received disease-related treatment; and (iii) without optimal cytoreductive surgery.

The treatment strategy was determined by a multidisciplinary gynecological oncology team. Preoperatively, all patients underwent an abdominopelvic clinical examination, chest computerized tomography (CT) and abdominal magnetic resonance imaging (MRI). If there was any doubt, positron emission tomography-computerized tomography (PET-CT) was performed to identify the suspicious metastases.

Patients were divided into two groups (1:1) according to a computer-generated permuted-block randomization, prepared by a statistician who was not involved in subsequent trial conduct. The random distribution cards were put into opaque envelopes. Enrolment and assignment were performed by a trial coordinator who was the only person able to access the locked, concealed randomization list. Patients were informed about the assignment method and the allocation outcomes. The informed consent was obtained from all patients included in this trial.

Patients in the control and experimental groups underwent infracolic and infragastric omentectomy respectively. Patients with intraoperative stage IA to IIA underwent hysterectomy, salpingooophorectomy, omentectomy, pelvic and para-aortic lymphadenectomy, peritoneal biopsy (uterovesical pouch, rectouterine pouch, both paracolic gutters and undersurfaces of diaphragm), aspiration of ascites or peritoneal lavage and removal of all visible diseases. Hysterectomy, salpingooophorectomy, omentectomy, suspicious and enlarged node resection, aspiration of ascites or peritoneal lavage and removal of all visible diseases were performed on patients with stage ≥ IIB. Infracolic omentectomy was defined as resection of the omentum under the level of the transverse colon. Infragastric omentectomy was defined as radical omtectomy including the vascular ring of the infragastric omental area. Laparoscopy will be first used to evaluate whether optimal cytoreduction can be achieved by a minimally invasive surgical approach. If not, an open procedure will be done. All surgeries were performed by an experienced certificated gynecological oncologist.

The excisional omentum in the experimental group was sent to pathologists in two parts, including the infracolic and infragastric omentum. If pathologists found no suspicious metastases on the omentum, 10 random samples were obtained to detect microscopic omental metastases [12]. All tissues were fixed in 4% neutralized formaldehyde, paraffin embedded, cut into sections, and stained with haematoxylin and eosin to confirm pathological diagnosis and other microscopic characteristics. The microscopic and macroscopic appearance of the omentum, including the size and location of metastasis was described. Furthermore, tumor size, depth of invasion, lymphatics, lymph node metastasis, and other metastases were also recorded.

The following clinical data of the patients were recorded such as age, body mass index (BMI), tumor markers, and International Federation of Gynecology and Obstetrics (FIGO) stage including intraoperative and final stage, surgical procedure, pathological results and complications. Patients received postoperative adjuvant platinum-based chemotherapy according to NCCN guidelines. Maintenance treatment (bevacizumab, polyadenosine diphosphate-ribose polymerase inhibition, or endocrine therapy) was not permitted. The follow up period was five years. The follow-up information was obtained from outpatient follow-up review once every three months in the first two years, every six months in the next three years and once a year thereafter. Telephone follow-up was performed once every six months. Relapse was defined as disease recurrence at any site. Disease recurrence was identified by imaging (e.g., MRI, PET-CT) and/ or with biochemically (e.g., elevated CA-125 levels). Imaging recurrence was assessed by RECIST v1.1. The primary endpoint was definitive comparison of PFS, which was defined as the time from randomization to death or recurrence, whichever occurred first. Prespecified secondary endpoints were omental metastasis detection rate, surgical complications, local recurrence rates and OS, which was defined as the time from randomization to death from any cause.

According to the previous research and our preliminary study, each arm used the hypothesis of a median progression free survival of 36 months and an alternative hypothesis of 65 months [13] With a type I error rate of 5% and a type II error rate of 20%, a target accrual of 47 patients per arm was planned. Thus, we aimed to recruit approximately 50 patients in each group. Statistical analyses were performed using SPSS for Windows 25.0 package. Continuous variables that complied with the normal distribution are reported as the average ± standard deviation; otherwise, these variables are reported as the median (quartile). Categorical variables are reported as absolute numbers (percentage). Comparisons between two groups of continuous variables were accomplished using independent samples t test or Mann–Whitney U test, as appropriate. Categorical covariates were compared with the chi-square test. A *P* value < 0.05 was considered statistically significant.

## Results

### Patient characteristics

**Fig. 1:**
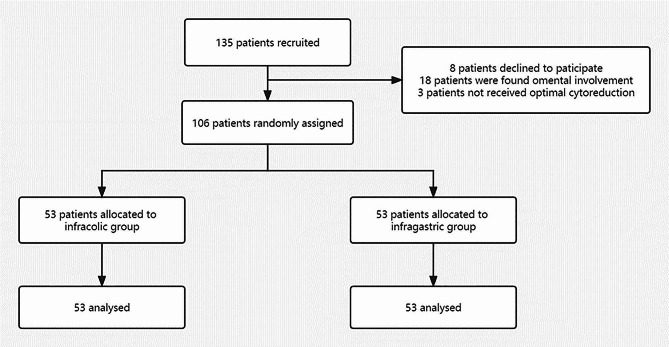
Participant flow of this study

In total, 135 patients were recruited and 106 patients who met the inclusion criteria were enrolled in this study. Of these, 53 patients underwent infracolic omentectomy, whereas 53 patients received infragastric omentectomy (Fig. 1). Patient characteristics are shown in Table 1. Most of characteristics in the infracolic and infragastric groups were similar. According to the analysis of the final stage, more patients in the infragastric group were diagnosed as FIGO stage III compared with the infracolic group, however the difference was not significant (*P* = 0.351).

**Table 1:** Patient characteristics

Patient characteristics	Infracolic group (*n* = 53)	Infragastric group (*n* = 53)	*P* value	
Age, mean ± SD (year)	51.5 ± 9.8	48.1 ± 8.6	0.058*	
BMI, median [quartile] (kg/m^2^)	22.1[19.9–24.6]	21.2[20.0-23.5]	0.446‡	
CA125, n(%)			0.151†	
<35U/ml	14(26.4)	8(15.1)		
≥35U/ml	39(73.6)	45(84.9)		
Surgical procedure, n (%)			0.587†	
Laparoscopy	46(86.8)	44(83.0)		
Laparotomy	7(13.2)	9(17.0)		
Ascites, n (%)			0.119†	
No	33(62.3)	25(47.2)		
Yes	20(33.7)	28(52.8)		
Intraoperative stage, n (%)			0.625†	
I	29(54.7)	27(50.9)		
II	15(28.3)	13(24.5)		
III	9(17.0)	13(24.5)		
Cytology, n (%)			0.169†	
Negative	34(64.2)	27(50.9)		
Positive	19(35.8)	26(49.1)		
Final stage, n (%)			0.351†	
I	27(50.9)	22 (41.5)		
II	12(22.6)	10(18.9)		
III	14(26.4)	21(39.6)		
Histologic type, n (%)				
High-grade serous	29(54.7)	30(56.6)	0.845†	
Low-grade serous	1(5.3)	1(5.3)		
Clear cell	8(15.1)	8(15.1)		
Endometrioid	8(15.1)	4(7.5)		
Mucinous	4(7.5)	10(18.9)		
Others	3(5.7)	0(0)		
Omentum involvement, n (%)			0.005†	
Yes	4(7.5)	15(28.3)		
No	49(92.5)	38(71.7)		
Lymphatic involvement, n (%)			0.780†	
Yes	7(13.2)	8(15.1)		
No	46(86.8)	45(84.9)		

*Independent-samples T test; Mann-Whitney U test; †Pearson chi-square test; SD: standard deviation

### Diagnostic value of infragastric omentectomy to tumor stage

In total, 36 patients were upstaged after the staging procedure or complete cytoreductive surgery. Of all, 19 patients were found to have omental metastatic lesions and their clinical characteristic details are shown in Table 2. Among them, 4 patients received infracolic omentectomy, whereas other patients underwent infragastric omentectomy. Seven patients changed their final stage due to newly found omental metastases, of which 6 patients were in the infragastric omentectomy group. Four patients in infragastric omentectomy group were intraoperatively evaluated as stage IIB, whereas the other patient was diagnosed as stage IA. These patients were upstaged to stage IIIA2 or IIIB after the infragastric omentectomy procedure. Only one patient in infracolic group was upstaged to stage IIIB from intraoperative stage IIB. Only one patient (No. 9) was diagnosed as mucinous adenocarcinoma, whereas other patients were high grade serous ovarian adenocarcinoma. She changed the adjuvant treatment recommendation after surgery according to the recent NCCN guidelines.

**Table 2:** Clinical characteristics of patients with omental metastases

No	Group	Intraoperative stage	Final stage	Histological type	Surgical procedure	Metastasis	
						Infracolic	Infragastric
1	Infragastric	IIB	IIIB	HGSOC	laparoscopy	0.2 cm	NA
2	Infragastric	IIIB	IIIB	HGSOC	laparoscopy	0.4 cm	NA
3	Infragastric	IIB	IIIA2	HGSOC	laparoscopy	NA	Microscopic
4	Infragastric	IIB	IIIA2	HGSOC	laparoscopy	Microscopic	NA
5	Infragastric	IIIC	IIIC	HGSOC	laparotomy	1 cm	NA
6	Infragastric	IIB	IIIB	HGSOC	laparotomy	1 cm	NA
7	Infragastric	IIIC	IIIC	HGSOC	laparoscopy	NA	0.4 cm
8	Infragastric	IIIB	IIIB	HGSOC	laparoscopy	Microscopic	Microscopic
9	Infragastric	IA	IIIB	Mucinous	laparoscopy	0.5 cm	0.5 cm
10	Infragastric	IIIB	IIIB	HGSOC	laparoscopy	0.8 cm	NA
11	Infragastric	IIIC	IIIC	HGSOC	laparoscopy	Microscopic	0.5 cm
12	Infragastric	IIIC	IIIC	HGSOC	laparoscopy	NA	Microscopic
13	Infragastric	IIIC	IIIC	HGSOC	laparoscopy	Microscopic	Microscopic
14	Infragastric	IIIC	IIIC	HGSOC	laparoscopy	Microscopic	Microscopic
15	Infragastric	IIB	IIIA2	HGSOC	laparoscopy	Microscopic	Microscopic
16	Infracolic	IIB	IIIB	HGSOC	laparoscopy	1 cm	Unknown
17	Infracolic	IIIB	IIIB	HGSOC	laparoscopy	0.8 cm	Unknown
18	Infracolic	IIIC	IIIC	HGSOC	laparotomy	1 cm	Unknown
19	Infracolic	IIIC	IIIC	HGSOC	laparotomy	Microscopic	Unknown

HGSOC: High grade serous ovarian carcinoma

In 15 patients in the infragastric group, omental metastatic lesions were found above transverse colon in 60.0% (9/15) of patients, whereas 20.0% (3/15) of them had no tumor below the transverse colon. The other 6 patients had omental metastatic lesions only below the transverse colon. The diameter of the metastatic lesion was no more than 1 cm.

### Factors predicting omental involvement

Factors predicting omental involvement in apparent early-stage epithelial ovarian cancer are shown in Table 3. According to the univariate analysis, significantly more omental involvement was found by pathologists in the infragastric group (78.9% vs. 21.0%, *P* = 0.009). Omental metastases were significantly related to involvement of other tumors such as the rectum, bladder and pelvic peritoneum (92.8% vs. 7.1%, *P* = 0.002). The omentum was more likely to be involved in high-grade serous adenocarcinoma than other histologic types (92.8% vs. 7.1%, *P* = 0.003). Compared with patients without omental metastases, more patients with omental involvement had ascites (68.4% vs. 31.6%, *P* = 0.030) and positive cytology (78.9% vs. 21.0%, *P* = 0.001).

**Table 3:** Factors predicating omental involvement in apparent early-stage epithelial ovarian carcinoma

	Univariate analysis			Multivariate analysis	
	No omental involvement (*n* = 87)	Omental involvement (*n* = 19)	*P* value	OR (95% CI)	*P* value
Age, mean ± SD (year)	49.7 ± 10.0	50.0 ± 8.1	0.912*	NA	NA
BMI, median [quartile] (kg/m^2^)	21.4[19.9–23.6]	21.8[19.9–25.2]	0.740†	NA	NA
CA125, median [quartile] (U/ml)	96.7[37.0-379.6]	327.8[113.3-710.5]	0.260†	NA	NA
Ascites, n(%)			0.030‡		
No	58(66.7)	6(31.6)		NA	NA
Yes	29(33.3)	13 (68.4)		NA	NA
Surgical procedure, n(%)			0.423§		
Laparoscopy	75(86.2)	15(78.9)		NA	NA
Laparotomy	12(13.8)	4(21.0)		NA	NA
Group, n(%)			0.009‡		
Infracolic group	49(56.3)	4(21.0)		1.000	
Infragastric group	38(43.7)	15(78.9)		6.519(1.765–24.079)	0.005
Cytology, n(%)			0.001‡		
Negative	57(62.3)	4(21.0)		NA	NA
Positive	30(37.7)	15(78.9)		NA	NA
Other involvement, n(%)			0.002‡		
No	50(57.5)	1(7.1)		1.000	
Yes	37(42.5)	18(92.8)		11.224(1.251-100.681)	0.031
Histologic type, n(%)			0.004‡		
HGSOC	41(47.1)	18(92.8)		1.000	
Others	46(52.9)	1(7.1)		0.117(0.013–1.079)	0.058

*Independent-samples T test; †Mann-Whitney U test; ‡Pearson chi-square test; §Chi-square test of continuity correction; SD: standard deviation; HGSOC: High grade serous ovarian carcinoma

Multivariate analysis showed that more patients in the infragastric group were diagnosed with omental metastases (OR: 6.519, *P* = 0.005). Moreover, other tumor involvements were also related to omental metastases (OR: 11.224, *P* = 0.031). Other factors such as histologic type and positive cytology were not correlated with omental involvement.

### Survival outcomes

**Fig. 2:**
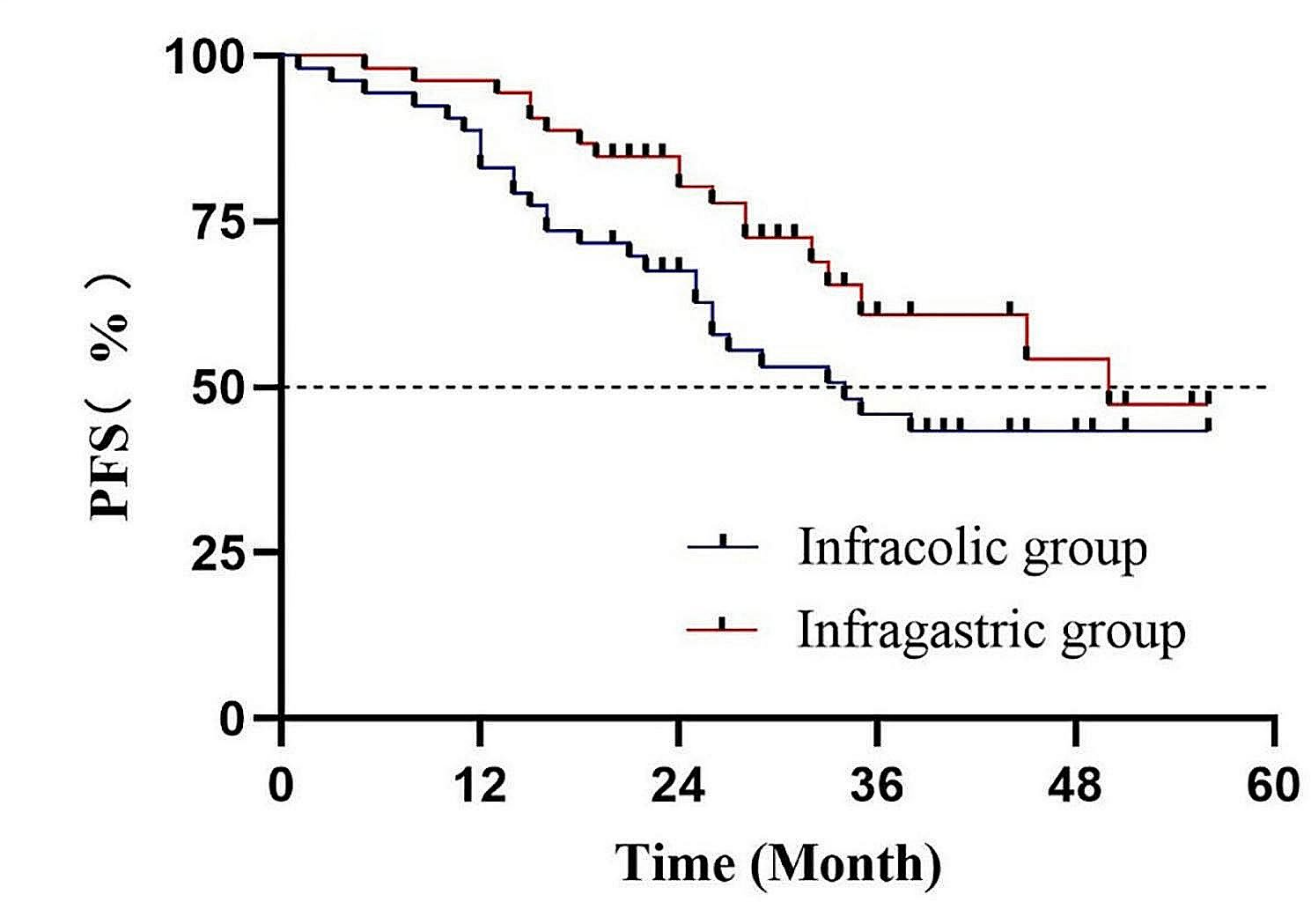
Progression-free survival according to the treatment group

Progression-free survival (PFS) did not show significant difference between two groups (*P* = 0.095). (Fig. 2) The median PFS in the infracolic and infragastric group was 34 months and 50 months, respectively. Twenty-seven patients in the infracolic group (50.9%) and 18 patients in the infragastric group (34.0%) had tumor recurrence during the follow-up (*P* = 0.077). Tables S1 and S2 showed clinical characteristics of patients with recurrence and the location of recurrent lesions in the two groups. The one-year recurrence rates were 16.98% (9/53) and 3.77% (2/53) in the infracolic and infragastric groups, respectively (*P* = 0.026). The two-year recurrence rates were 36.17% (17/47) and 19.56% (9/46) in the infracolic and infragastric group (*P* = 0.074). Among 27 patients with recurrent tumor in the infracolic group, recurrent lesions on residual infragastric omentum were diagnosed in 15 patients. Moreover, more recurrent patients in the infracolic group had upper abdominal metastatic lesions compared with those in the infragastric group (20 cases vs. 8 cases *P* = 0.045). More patients in the infragastric group received secondary cytoreductive surgery after tumor recurrence (*P* = 0.020). Up to March 2023, 4 patients in the infracolic group and 2 patients in the infragastric group died due to tumor progression.

**Fig. 3:**
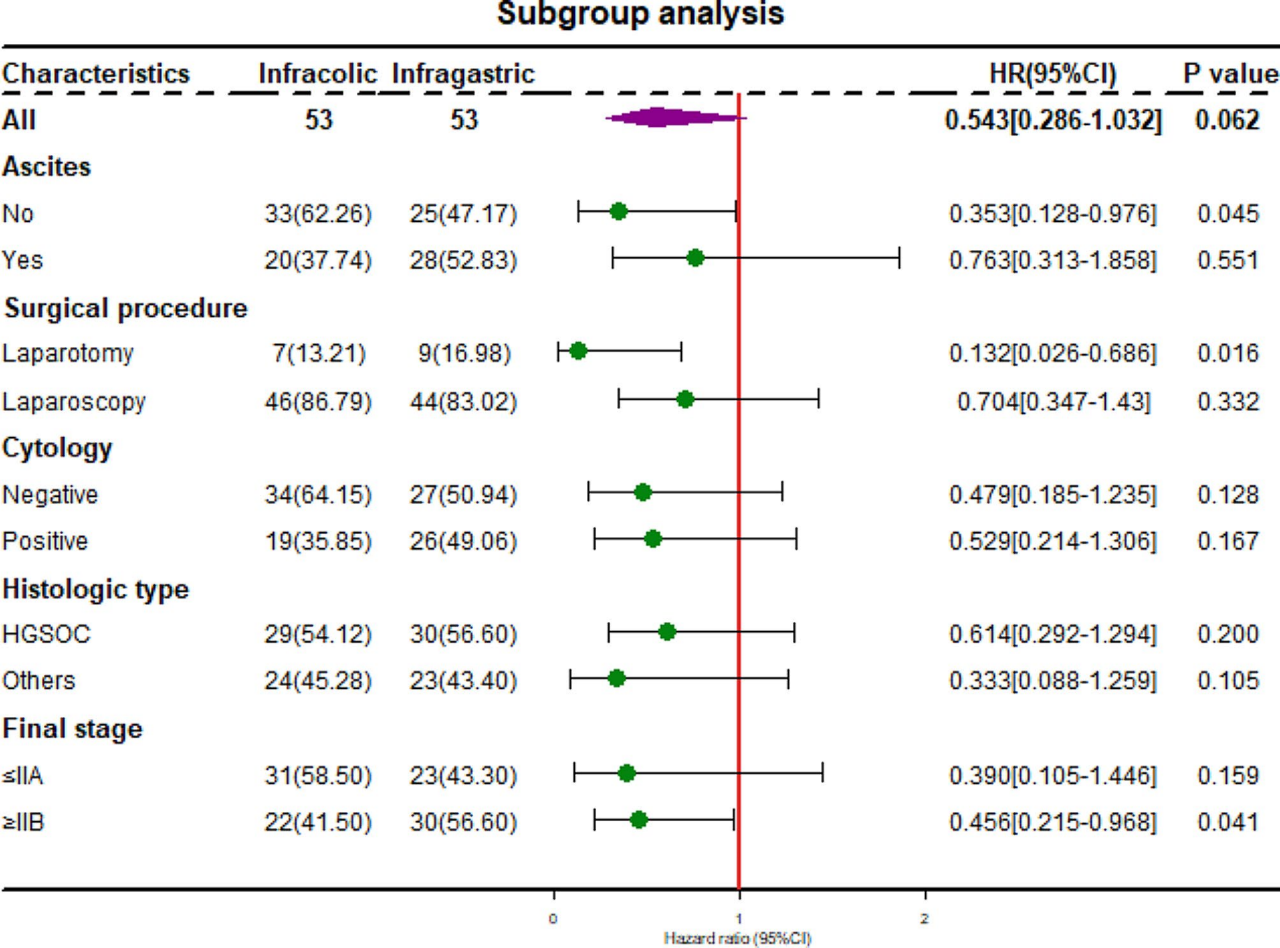
Treatment effect on PFS by subgroup. HR < 1 showed favouring infragastric omentectomy

Subgroup comparisons for PFS of the subgroups which divided by ascites, surgical procedure, cytology, histologic type, and final stage are shown in Fig. 3. Patients with final stage IIB-IIIC (HR: 0.456, *P* = 0.041), undergoing laparotomy (HR: 0.132, *P* = 0.016) and without ascites (HR: 0.353, *P* = 0.045) showed a benefit from infragastric omentectomy. Cox regression analysis in patients with final stage IIB-IIIC was showed in table S3, which indicated that infragastric omentectomy is related to longer PFS (*P* = 0.002).

### Surgical complications

The surgical characteristics and survival outcomes are shown in Table 4. Extending the scope of surgery may lead to more injuries and bleeding. More patients received blood transfusions in the infragastric group; However, the difference was not significant (15.1% vs. 13.2%, *P* = 0.780). The estimated blood loss in the infragastric group was similar to that in the infracolic group (200 ml vs. 200 ml, *P* = 0.771). The operation time in infragastric group was slightly longer than that in the infracolic group (210–300 min vs. 193.5–292.5 min, *P* = 0.481). The two groups had similar times of antibiotic treatment (5 d vs. 5 d, *P* = 0.475), hospital stay (11 d vs. 11 d, *P* = 0.367), delivery of the first chemotherapy (18.5 d vs. 17 d, *P* = 0.942) and postoperative fasting (3d vs. 3d, *P* = 0.385). Two patients in the infracolic group and 1 patient in the infragastric group developed intestinal obstruction during the follow-up period.

**Table 4:** Perioperative surgical characteristics

	Infracolic group (*n* = 53)	Infragastric group (*n* = 53)	*P* value
Operating time, median [quartile] (min)	240[193.5-292.5]	240[210–300]	0.481*
Estimated blood loss, median [quartile] (ml)	200[100–300]	200[100–200]	0.771*
Hospital stays, median [quartile] (day)	11[8-14.5]	11[9–15]	0.367*
Time to delivery of adjuvant therapy, median [quartile] (day)	18.5[14-22.5]	17[15–21]	0.942*
Time of antibiotic treatment, median [quartile] (day)	5[4–6]	5[4–6]	0.475*
Postoperative fasting, median [quartile] (day)	3[2–3]	3[2–4]	0.385*
Blood Transfusion, n (%)			0.780†
Yes	7(13.2)	8(15.1)	
No	46(86.8)	45(84.9)	

*Mann-Whitney U test; †Chi-square test of continuity correction

## Discussion

The omentum is a primary site of metastatic spread in advanced stage epithelial ovarian cancer [14]. Optimal cytoreductive surgery can significantly improve patient survival [15]. In addition, animal experiments showed that surgery can affect the genetics dictating the microenvironment of metastatic ovarian cancer [16]. Even without intraoperative omental findings, isolated omental metastases were found in 2–7% of patients with early-stage epithelial ovarian cancer [5]. Patients with early-stage ovarian cancer typically receive infracolic omentectomy as a staging surgery. Extending the scope of the omentum may reduce the omission of metastases and improve diagnosis and prognosis. This research compared the benefit and risk of patients who received different levels of omentectomy.

As previously reported, ascites, positive cytology, high intraoperative stage, and other tumor involvement may be associated with several high risks of omental involvement [17–21]. Our research also showed the relationship between omental involvement and several characteristics such as positive cytology, high-grade serous adenocarcinoma and other tumor involvement. Possibly due to our limited sample size, high-grade serous adenocarcinoma did not show a significant difference in relation to omental metastasis based on the multivariate analysis.

As a traditional procedure of staging surgery, omentectomy plays an important role in the stage of ovarian cancer [18]. According to our results, omental metastasis was a main cause that led to a higher tumor stage. Our study indicated that more omental metastases were found in infragastric omentectomy than in infracolic omentectomy. Infragastric omental metastases were found in 9 patients, indicating that infragastric omentectomy reduces the residual lesion effectively. Six patients changed their tumor stage from stage IIB to stage IIIA2 or IIIB, indicating that patients with pelvic metastasis were more likely to have involvement of omentum.

Compared with previous studies, the proportion of patients who were upstaged due to omental metastasis in this study was slightly higher, which may be related to the extended resection area of the omentum in this study [22]. After excluding the effect of other risk factors, significantly more omental metastases were found by pathologists in the infragastric group. Compared with infracolic omentectomy, infragastric omentectomy seemed to be more accurate in tumor staging.

In a study including 24 cases with advanced ovarian cancer who had no apparent infragastric omental involvement, all patients received infragastric omentectomy [23]. The postoperative pathologic findings showed that 15 patients (62.5%) had micrometastases in the infragastric omentum. Among those cases, 10 also had infracolic metastases, whereas the transverse colon was not involved in the remaining 5 cases. The rate of omental involvement in our study was slightly lower because patients with early-stage disease were included. This study also indicated that not only the infracolic omentum but also the infragastric omentum can only be involved in tumor metastases microscopically. For those patients without apparent omental metastasis, infragastric omentectomy also played an important role in staging or cytoreductive surgery. If only infracolic omentectomy was performed, approximately 11.3% of patients (6/53) may have minimal residual disease on the infragastric omentum, which may affect the postoperative diagnosis and prognosis. In the infragastric group, (5/26) 19.2% of patients with intraoperative stage II or III had infragastric omental involvement. Moreover, the status of one patient in our study (No.3) changed her stage to from IIB to IIIB because of isolated infragastric omental metastasis. If the extension of the omentectomy her received was insufficient in this patient, the patient could be possibly diagnosed as stage IIB by mistake.

Our research showed that metastases can be found on the infragastric omentum, even without infracolic omental involvement, especially for patients with intraoperative stage II or III disease. Residual microscopic disease on infragastric omentum may be omitted if patients only underwent infracolic omentectomy. If those metastases were omitted, tumor stage and prognosis may be affected. As a result, resection of the infragastric omentum may seem necessary for patients with intraoperative stage II or III disease.

Satisfactory cytoreductive surgery is one of the most important factors for the prognosis of patients with advanced ovarian cancer [24, 25]. A previous study showed that among patients after satisfactory cytoreductive surgery, patients without micrometastases had a better prognosis compared with those who had micrometastases according to pathological examination [26]. In addition, according to recent NCCN guidelines, some stage I patients could choose observation instead of receiving adjuvant chemotherapy [6]. If they were diagnosed as a lower stage by mistake, they might miss the perfect time to receive chemotherapy which could lead to a worse prognosis. It was demonstrated in animal experiments that residual micrometastases in the omentum will eventually progress if further adjuvant treatment is not administered [27]. In a previous retrospective study of 256 patients with normal-appearing omentum, one patient received a further chemotherapy based on microscopic omental metastasis [21]. However, in another study, no patients changed to suggested treatment based on microscopic involvement of the omentum [28]. In this research, a patient (No. 9) with intraoperative stage I mucinous adenocarcinoma was diagnosed with final stage IIIB. As a result, she changed her adjuvant treatment recommendation after surgery.

According to a previous study including 57 patients diagnosed with complete remission after treatment, the area of resected omentum was not an independent risk factor for tumor relapse [29]. Compared with infracolic omentectomy, our research showed the infragastric omentectomy can improve the PFS of patients, although the difference is not significant. More patients in the infracolic group had tumor recurrence especially in the first year. In addition, more patients in the infracolic group died due to tumor progression. This finding indicated that infragastric omentectomy might be beneficial to the prognosis of patients who met our inclusion criteria.

Epithelial ovarian cancer is widely believed to metastasize via direct surface spread according to the “soil and seeds” theory [30]. However, previous research showed that ovarian cancer can also metastasize to the omentum by haematogenous dissemination [31]. The omentum is one of the most common recurrent sites of ovarian cancer given its nutrient supply for cancer cells [16, 32, 33]. In our research, more than half of the patients (15/27) with residual infragastric omentum had recurrent lesions on the omentum. This result was similar to that noted in previous research [16, 32, 33].

Moreover, residual infragastric omentum seemed to be related to upper abdominal metastases. Adipocytes in the omentum may promote ovarian cancer metastasis and provide energy for rapid tumor growth [11, 34, 35, 11] Complete resection of recurrent metastases in the upper abdomen is one of the main difficulties encountered during secondary cytoreductive surgery. Our study indicated that extending omentectomy may significantly decrease recurrence in the upper abdomen so that they could be more likely to receive optimal secondary cytoreduction.

According to the subgroup analysis, patients with final stage IIB-IIIC showed a benefit from infragastric omentectomy. Patients with advanced stage disease have a higher risk of recurrence. Extended excision of omentum can improve the prognosis via reducing the recurrence especially in the upper abdomen. If suspicious metastases out of the pelvic cavity were found intraoperatively, infragastric omentectomy was preferred instead of infracolic one. However, more related researches should be carried out in the future. What’s more, laparotomy was applied to patients with higher tumor burden. All patients who received laparotomy were intraoperative stage III. This result indicated that the higher stage was diagnosed, the better prognosis was given by the infragastric omentectomy.

Excluding the benefit of diagnosis and treatment to the patients, complications of extending omentectomy were also discussed in our research. Infracolic omentectomy was considered to have “almost no complications” [36]. The most common complications associated with expanding the scope of surgery are prolonged operation time and increased intraoperative blood loss. Postoperative complications will also cause a prolonged hospital stay and delayed chemotherapy. EOC patients who underwent cytoreductive surgery had longer hospital stays and more complications, especially those whose surgical scope involved the upper abdomen [37]. Due to the extra excisional area, the operating time in the infragastric group was slightly longer. However, hospital discharge or first chemotherapy was not delayed because of infragastric omentectomy.

Additionally, the omentum is known to defend the peritoneal cavity against infections and wall off foreign bodies [38, 39]. Enlarging the resection area of the omentum may cause postoperative infection in patients. Animal experiments also confirmed that the omentum plays an important role in the peritoneal defence system. If the greater omentum is removed, the risk of bacteria entering the bloodstream increases, and peritoneal infections may be more serious [40]. In this study, a similar period of antibiotic treatment showed that excision of the infragastric omentum did not increase the risk of infection in the short term. In addition, cutting off gastroepiploic vessels, in infragastric omentectomy may impact the blood supply and recovery of the stomach after surgery. However, no significant difference in the postoperative fasting was noted between the two groups. Therefore, we believe that the risk of infragastric omentectomy is relatively limited. After training and learning, gynecologists can complete the procedure independently [41].

The current study has several limitations. First, although no difference between laparoscopy and laparotomy in predicting omental involvement in apparent early-stage epithelial ovarian cancer was found, it would be more convincing if the same surgical procedure was performed. Second, although several positive results were found in our research, our sample size was still limited at both arms. These findings remain to be confirmed in a larger population study. Moreover, the long-term complications and overall survival were not evaluated because of the insufficient follow-up period. We continued to follow up the patients and evaluate the long-term survival.

## Conclusions

This study is the first to compare the benefits and risks of different types of omentectomy for patients with epithelial ovarian cancer. Compared with infracolic omentectomy, a traditional part of staging surgery, more omental metastases could be discovered by infragastric omentectomy without more complications. Infragastric omentectomy was a more appropriate surgical procedure which can improve the PFS and decrease tumor relapse in the upper abdomen especially for patients with stage IIB-IIIC disease. However further large-scale and long-term prospective investigations are required to address this interesting and important issue.

### Electronic supplementary material

Below is the link to the electronic supplementary material.

Supplementary Material 1

## Data Availability

The datasets generated and/or analysed during the current study are not publicly available due to uncompleted clinical trial but are available from the corresponding author on reasonable request.

## Abbreviations

PFS: Progression-free survival
OS: Overall survival
CT: Computerized tomography
MRI: Magnetic resonance imaging
PET-CT: Positron emission tomography-computerized tomography
SD: Standard deviation
HGSOC: High grade serous ovarian cancer
