# Supplementary material for: A randomized controlled trial to compare short-term outcomes following infragastric and infracolic omentectomy at the time of primary debulking surgery for epithelial ovarian cancer with normal-appearing omentum
Source: J Ovarian Res. 2024 Apr 19;17:85. doi: 10.1186/s13048-024-01401-8 (PMC11027406; doi:10.1186/s13048-024-01401-8)
Supplement: Supplementary file 1 — Supplementary Material 1 [file 13048_2024_1401_MOESM1_ESM.docx]

Table S1. Clinical characteristics of patients with recurrence

| No | Group | Final stage | Histological type | Diagnostic basis | Omental relapse | Upper abdominal relapse | Treatment | Death |
| --- | --- | --- | --- | --- | --- | --- | --- | --- |
| 1 | Infragastric | IIIC | HGSOC | Radiography | / | Yes | Chemotherapy | No |
| 2 | Infragastric | IIIC | HGSOC | Pathology | / | Yes | Surgery+ Chemotherapy | No |
| 3 | Infragastric | IIIB | HGSOC | Pathology | / | No | Surgery+ Chemotherapy+ PARPi | No |
| 4 | Infragastric | IIIB | HGSOC | Radiography | / | No | Chemotherapy | No |
| 5 | Infragastric | IIIB | HGSOC | Pathology | / | No | Surgery+ Chemotherapy | No |
| 6 | Infragastric | IC3 | HGSOC | Pathology | / | No | Chemotherapy | No |
| 7 | Infragastric | IIIA2 | HGSOC | Pathology | / | Yes | Surgery+ Chemotherapy | No |
| 8 | Infragastric | IA | Clear cell | Pathology | / | No | Surgery+ Chemotherapy+PARPi | No |
| 9 | Infragastric | IIIB | HGSOC | Pathology | / | No | Surgery+ Chemotherapy+PARPi | No |
| 10 | Infragastric | IIB | HGSOC | Radiography | / | Yes | Chemotherapy | Yes |
| 11 | Infragastric | IC2 | Clear cell | Radiography | / | Yes | Chemotherapy | No |
| 12 | Infragastric | IIIB | Mucinous | Radiography | / | Yes | Chemotherapy | No |
| 13 | Infragastric | IIIB | HGSOC | Pathology | / | No | Surgery+ Chemotherapy+PARPi | No |
| 14 | Infragastric | IIIC | HGSOC | Pathology | / | No | Surgery+ Chemotherapy+PARPi | No |
| 15 | Infragastric | IIIC | HGSOC | Radiography | / | Yes | Chemotherapy | No |
| 16 | Infragastric | IIB | HGSOC | Pathology | / | Yes | Surgery+ Chemotherapy+PARPi | No |
| 17 | Infragastric | IIB | HGSOC | Pathology | / | No | Surgery+ Chemotherapy+PARPi | No |
| 18 | Infragastric | IIIC | HGSOC | Radiography | / | Yes | Chemotherapy | No |
| 19 | Infracolic | IC2 | Mucinous | Radiography | No | Yes | Chemotherapy | No |
| 20 | Infracolic | IIIA1ii | HGSOC | Radiography | Yes | Yes | Chemotherapy | Yes |
| 21 | Infracolic | IIIB | HGSOC | Pathology | No | Yes | Surgery+ Chemotherapy | No |
| 22 | Infracolic | IIIB | HGSOC | Pathology | Yes | Yes | Chemotherapy | No |
| 23 | Infracolic | IIIB | HGSOC | Radiography | No | Yes | Chemotherapy | No |
| 24 | Infracolic | IC3 | Clear cell | Radiography | Yes | Yes | Chemotherapy | Yes |
| 25 | Infracolic | IC1 | Clear cell | Radiography | No | Yes | Chemotherapy | No |
| 26 | Infracolic | IIIB | HGSOC | Radiography | Yes | Yes | Chemotherapy | Yes |
| 27 | Infracolic | IC3 | Clear cell | Pathology | No | Yes | Surgery+ Chemotherapy | No |
| 28 | Infracolic | IC3 | HGSOC | Pathology | Yes | Yes | Surgery+ Chemotherapy | No |
| 29 | Infracolic | IIA | HGSOC | Radiography | Yes | Yes | Chemotherapy | No |
| 30 | Infracolic | IIIB | HGSOC | Pathology | Yes | Yes | Surgery+ Chemotherapy | No |
| 31 | Infracolic | IIIC | HGSOC | Pathology | Yes | Yes | Surgery+ Chemotherapy | Yes |
| 32 | Infracolic | IIB | HGSOC | Pathology | No | No | Surgery+ Chemotherapy+PARPi | No |
| 33 | Infracolic | IIIC | HGSOC | Pathology | Yes | Yes | Surgery+ Chemotherapy+PARPi | No |
| 34 | Infracolic | IC1 | Endometrioid | Pathology | No | No | Surgery+ Chemotherapy | No |
| 35 | Infracolic | IIIB | HGSOC | Pathology | No | Yes | Surgery+ Chemotherapy | No |
| 36 | Infracolic | IIIA1ii | HGSOC | Pathology | Yes | Yes | Surgery+ Chemotherapy+PARPi | No |
| 37 | Infracolic | IC1 | Endometrioid | Pathology | No | No | Surgery+ Chemotherapy+PARPi | No |
| 38 | Infracolic | IIB | HGSOC | Pathology | No | No | Surgery+ Chemotherapy+PARPi | No |
| 39 | Infracolic | IIB | HGSOC | Pathology | Yes | Yes | Surgery+ Chemotherapy+PARPi | No |
| 40 | Infracolic | IIB | HGSOC | Pathology | Yes | Yes | Surgery+ Chemotherapy+PARPi | No |
| 41 | Infracolic | IIIC | HGSOC | Radiography | Yes | Yes | Chemotherapy | No |
| 42 | Infracolic | IIIA2 | Clear cell + Endometrioid | Radiography | Yes | Yes | Chemotherapy | No |
| 43 | Infracolic | IC2 | Clear cell | Pathology | No | No | Surgery+ Chemotherapy+PARPi | No |
| 44 | Infracolic | IC2 | Mucinous | Radiography | Yes | Yes | Chemotherapy | No |
| 45 | Infracolic | IIIA2 | Endometrioid | Pathology | No | No | Surgery+ Chemotherapy+PARPi | No |

HGSOC: High grade serous ovarian carcinoma

Table S2. Location of recurrent lesions in different groups

|  | Infracolic (n=27) | Infragastric (n=18) |
| --- | --- | --- |
| Pelvic peritoneum | 23 | 14 |
| Abdominal peritoneum | 18 | 11 |
| Retroperitoneal lymph node | 4 | 5 |
| Rectum | 5 | 3 |
| Colon | 7 | 4 |
| Small intestine | 5 | 3 |
| Bladder | 6 | 1 |
| Omentum | 15 | / |
| Mesentery | 11 | 5 |
| Spleen | 7 | 3 |
| Liver | 6 | 3 |
| Pancreas | 4 | 1 |
| Cholecyst | 2 | 1 |
| Stomach | 1 | 1 |
| Diaphragm | 14 | 8 |
| Distant metastases | 6 | 8 |

Table S3: Cox regression analysis in patients with final stage IIB-IIIC

|  | | Univariate analysis | | | Multivariate analysis | |
| --- | --- | --- | --- | --- | --- | --- |
|  | HR (95% CI) | | P value | HR (95% CI) | | P value |
| Age | | 1.058(0.989-1.071) | 0.158 | NA | | NA |
| BMI | | 1.135(0.983-1.309) | 0.083 | NA | | NA |
| CA125 | | 1.000(1.000-1.001) | 0.148 | NA | | NA |
| Group | |  |  |  | |  |
| Infracolic | | 1.000 |  | 1.000 | |  |
| Infragastric | | 0.490 (0.243-0.991) | 0.047 | 0.307(0.144-0.655) | | 0.002 |
| Ascites | |  |  |  | |  |
| No | | 1.000 |  | NA | | NA |
| Yes | | 1.588 (0.775-3.253) | 0.206 | NA | | NA |
| Cytology | |  |  |  | |  |
| Negative | | 1.000 |  | NA | | NA |
| Positive | | 1.618(0.806-3.250) | 0.176 | NA | | NA |
| Histologic type | |  |  |  | |  |
| Others | | 1.000 |  | NA | | NA |
| HGSOC | | 2.952(0.897-9.793) | 0.076 | NA | | NA |
| Final stage | |  |  |  | |  |
| Stage IIB | | 1.000 |  | 1.000 | |  |
| Stage III | | 3.650(1.476-9.026) | 0.005 | 5.990(2.220-16.158) | | <0.001 |
